# Study on the Cytotoxic Activity of Drimane Sesquiterpenes and Nordrimane Compounds against Cancer Cell Lines

**DOI:** 10.3390/molecules191118993

**Published:** 2014-11-18

**Authors:** Ivan Montenegro, Giacomo Tomasoni, Claudia Bosio, Natalia Quiñones, Alejandro Madrid, Hector Carrasco, Andres Olea, Rolando Martinez, Mauricio Cuellar, Joan Villena

**Affiliations:** 1 Facultad de Farmacia, Escuela de Química y Farmacia, Universidad de Valparaíso, Av. Gran Bretaña N° 1093, Valparaíso 234000, Chile; E-Mails: ivan.montenegro@uv.cl (I.M.); natuka_q@hotmail.com (N.Q.); 2 Departamento de Química, Universidad Técnica Federico Santa María, Av. España N° 1680, Valparaíso 2340000, Chile; E-Mail: alejandro.madrid@usm.cl; 3 Departamento de Química, Facultad de Ciencias Exactas, Universidad Andrés Bello, Quillota 910, Viña del Mar 2520000, Chile; E-Mails: volgin_4@hotmail.com (G.T.); samybrs@hotmail.com (C.B.); rmartinez@unab.cl (R.M.); 4 Facultad de Ciencias de la Salud, Universidad Autónoma de Chile, Carlos Antúnez 1920, Providencia, Santiago 7500000, Chile; E-Mails: hector.carrasco@uautonoma.cl (H.C.); andres.olea@uautonoma.cl (A.O.); 5 Centro de Investigaciones Biomédicas (CIB), Escuela de Medicina, Universidad de Valparaíso, Av. Hontaneda N° 2664, Valparaíso 234000, Chile

**Keywords:** cytotoxic activity, cancer cell lines, apoptosis, mitochondrial membrane permeability, caspasa-3 activity, nordrimanes, drimanes, sesquiterpenes

## Abstract

Twelve drimanes, including polygodial (**1**), isopolygodial (**2**), drimenol (**3**), confertifolin (**4**), and isodrimenin (**5**), were obtained from natural sources. Semi-synthetic derivatives **6**–**12** were obtained from **1** and **2**, and cytotoxic activity was evaluated *in vitro* against cancer cell lines (HT-29, MDA-MB231, DHF, MCF-7, PC-3, DU-145, and CoN). IC_50_ values were determined at concentrations of 12.5–100 µM of each compound for 72 h. In addition, it was found that polygodial (**1**), **8**, and **12** induced changes in mitochondrial membrane permeability in CoN, MCF-7, and PC-3 cells.

## 1. Introduction

The third leading cause of cancer-related deaths in women, breast cancer incidence has increased worldwide, although this may be in part due to improvements in early detection. Just as for breast cancer in women, prostate cancer is the most common non-skin malignancy in men. In the United States, for example, there were an estimated 244,000 new cases and 40,400 deaths from this cause in 1995 alone. In Chile, the risk of death from this disease has tripled in the last 40 years; the figures for 1998 indicate 1,218 deaths were recorded from prostate cancer in the country, determining a mortality rate of 16.6 per 100,000 men [1]. In Chile, prostate cancer is the second leading cause of cancer death in men, similar to developed countries. Despite the high prevalence and mortality, there are no established screening programs for early detection [2]. Due to this need, several drugs with anticancer effects have been extracted from plants in the last 20 years, many of which are particularly effective against breast cancer cells [3].

*Drymis winteri* Forst (Canelo) has been used in Chilean folk medicine in the treatment of inflammatory diseases [4], against tumors, uterine fibromas, malignant ulcers, and for its antifungal properties. Several sesquiterpenes have been found in *Drymys* species, such as polygodial (**1**), isopolygodial (**2**), drimenol (**3**), confertifolin (**4**), and isodrimenin (**5**) [5]. Polygodial possesses a wide range of potential biological applications, including antibacterial [6], anti-allergic and anti-inflammatory [7], and antifungal [8].

As part of a general attempt to study structure-activity relationships for unsaturated dialdehydes from natural sources, several compounds have been investigated using the *Salmonella*-microsome assay (strains TA 98, TA 2637, and TA 100). Polygodial and isopolygodial showed no mutagenic activity at their highest non-toxic concentration [9]. Furthermore, unsaturated sesquiterpene dialdehydes were tested for antimicrobial, cytotoxic, and mutagenic activity. Where Polygodial exhibited antibacterial and cytotoxic activity, isopolygodial was slightly less active [10].

Although the claims that **1** possesses cytotoxic activity were consistent with previous reports [11,12,13,14], contradictory reports were found in the literature regarding the cytotoxic activities of its natural analogue **2**, which was variously reported to be either active or inactive [15].

In the course of our search for bioactive compounds from natural sources, we recently reported the antifungal and antifeedant activity of naturally occurring drimanes and their semi-synthetic derivatives, including polygodial, isopolygodial, drimenol, and confertifoline [15,16]. In order to clarify the main features required for drimanes to display cytotoxic activities, we tested herein a series of 12 compounds **1**–**12** (most of them not previously assayed for cytotoxic activities) for antitumoral properties against a unique panel of five cancer cell lines. 

## 2. Results and Discussion

### 2.1. Chemistry

**Figure 1:**
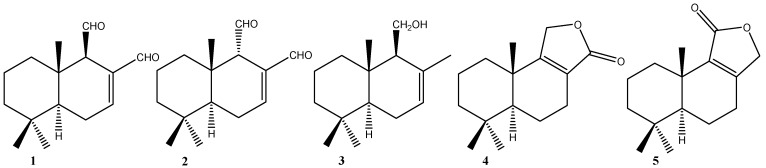
Structure of natural drimanes from bark of the *D. winteri*.

Sesquiterpenes **1**–**5** (Figure 1) were obtained from *D. winteri* [16]; compounds **6**–**8** were obtained from polygodial; and **9**–**12**, from drimenol [16,17]. 

#### 2.1.1. Compounds Obtained from **1**

**Scheme 1:**
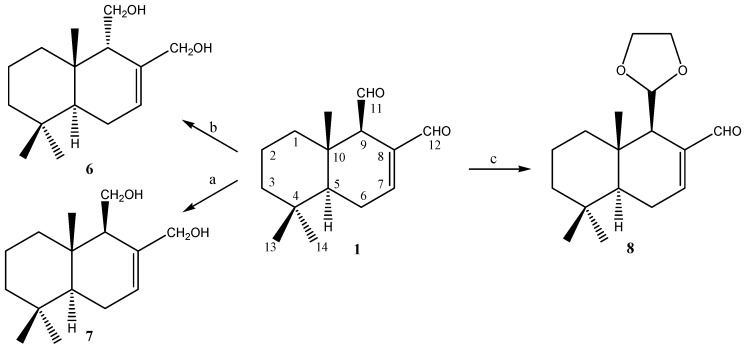
Modifications performed on **1** to obtain compounds **6**–**8**.

Scheme 1 shows the modifications performed on compound **1** that led to compounds **6**–**8**. Reduction of **1** with NaBH_4_, in MeOH and at room temperature, produced 9β-drimendiol **7**. Following this, when **1** was epimerized at C-9, with 5% NaOH in MeOH solution, and subsequently reduced with NaBH_4_ in MeOH at room temperature, 9α-drimendiol **6** was obtained. When **1** was treated with one equivalent of ethylene glycol in benzene, in the presence of a catalytic amount of *p*-toluenesulfonic acid, monoacetal **8** was produced.

*Conditions and reagents*: **a**. NaBH_4_ (excess), MeOH rt; **b**. KOH, MeOH (50%), NaBH_4_ (excess), MeOH rt; **c**. p-TosOH, ethylene glycol.

#### 2.1.2. Compounds Obtained from **3**

**Scheme 2:**
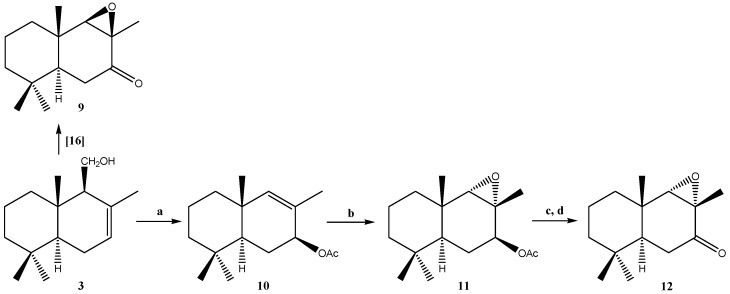
Modifications performed on **3** to obtain compounds **9**–**12**.

Scheme 2 shows the modifications performed on compound **3**. In this respect, we recently reported the synthesis of epoxinordrimane **9** [17]. In order to allow the synthesis of isomer **12**, we proceeded to treat **3** with LDA in refluxing benzene, which allowed us to obtain 11-nordrimanic acetate **10**. 

*Conditions and reagents*: **a**. LTA (lead IV acetate), PhH, Reflux; **b**. m-CPBA, CH_2_Cl_2_, r.t, 1.5 h; **c**. MeONa, MeOH, N_2_, r.t, 2 h; **d**. PDC, CH_2_Cl_2_, N_2_, r.t, 1.5 h.

The subsequent epoxidation of **10** with *m*-chloroperbenzoic acid (MCPBA) produced epoxide **11**. Subsequent hydrolysis of **11** under alkaline conditions and posterior oxidation with PCC in dichloromethane lead to epoxinordrimane **12**. The identities of **6**, **7**, and **8** were established through comparisons with previously reported data [16]. Compounds **11** and **12**, which were only previously reported once, are described in detail in this work.

**Figure 2:**
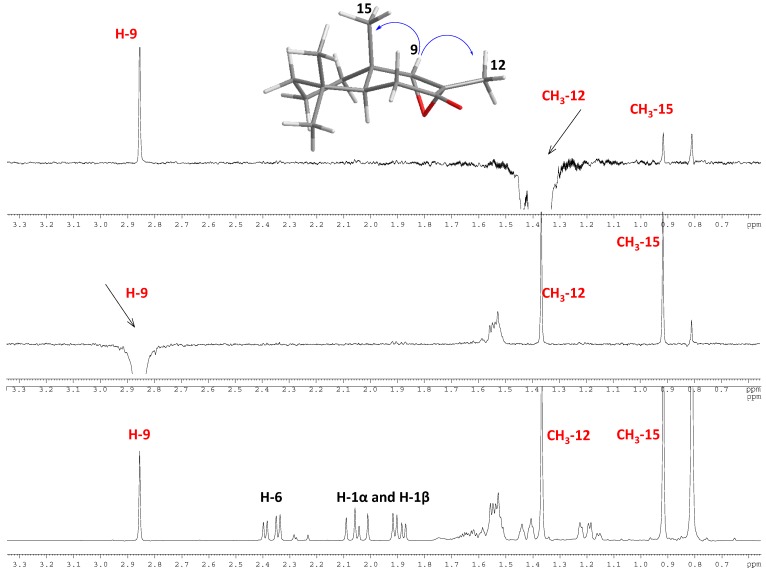
Bottom: ^1^H-NMR spectrum of compound **12**. Top: 1D ^1^H NOESY experiment (*selnogp* pulse program from Bruker Library) showing the main long-range interactions between CH_3_-12 and H-9, H-9 and CH_3_-15.

We recently reported a derivative series from drimenol and polygodial [15]. The assignment of the configuration in C-8 and C-9 for compound **12** was proposed on the basis of NOE-spectra observations. From a ^1^H-1D -NOESY experiment, when CH_3_-12 was selectively irradiated, only long-range interactions with H-9 and CH_3_-15 were observed. In addition, when H-9 was selectively irradiated, a long-range interaction with CH_3_-15 was observed. NMR experiments thus confirm the α spatial orientation of the epoxide ring and the stereochemistry of C-8 and C-9, as shown in Figure 2.

### 2.2. Biological Results

Previous programs designed to discover more active compounds from *D. winteri* and its derivatives, had yielded results suggesting that these compounds showed cytotoxic activity. Based on that information, and knowing that this tree has high activity potential metabolites, a growth regulatory study on cancer cell lines has been carried out. 

### 2.3. Viability Assay

The cytotoxicity of compounds **1**–**12** was evaluated *in vitro* against different cancer cell lines: MCF-7 breast cancer, DU-145 and PC-3 human prostate cancer, and one non-tumor cell line, human colon epithelial cells CCD 841 CoN (CoN). A colorimetric assay was set up to estimate the IC50 values, which represent the concentration of a drug that is required for 50% inhibition *in vitro* after 72 h of continuous exposure to the test compounds. Four serial dilutions (from 12.5 to 100 μM) for each sample were evaluated in triplicate. The IC_50_ obtained from these assays are shown in Table 1. 

**Table 1:** Cytotoxicity (IC50 μM) of compounds **1**–**12**.

Compound		DU145	PC-3	MCF-7	CoN
**1**	**IC_50_**	**71.4 ± 8.5**	**89.2 ± 6.8**	**93.7 ± 9.1**	>200
**2**	**IC_50_**	>200	>200	>200	>200
**3**	**IC_50_**	>200	>200	>200	>200
**4**	**IC_50_**	>200	>200	>200	>200
**5**	**IC_50_**	**90.5 ± 8.2**	**87.6 ± 9.2**	>200	>200
**6**	**IC_50_**	>200	>200	>200	>200
**7**	**IC_50_**	>200	>200	>200	>200
**8**	**IC_50_**	**70.6 ± 5.9**	**65.4 ± 5.5**	**97.1 ± 7.2**	>200
**9**	**IC_50_**	**93.5 ± 6.7**	**97.5 ± 10.4**	>200	>200
**10**	**IC_50_**	>200	>200	>200	>200
**11**	**IC_50_**	>200	>200	>200	>200
**12**	**IC_50_**	>200	**90.2 ± 8.8**	**88.4 ± 7.1**	>200

The highest cytotoxicity values were observed for polygodial (**1**) and compound **8** in all cell lines tested and were more active than those of the rest of compounds. The cytotoxicity of compounds in colon epithelial cells (CoN) is lower than in the cancer cell lines under study, indicating that the compounds are some selectivity to cancer cells.

Since compounds **1**, **8** and **12** had inhibitory effects on the growth of the cancer cell types tested, the effect of these compounds required study in greater detail. Moreover, the compounds **1** and **8** associated with α,β-unsaturated systems and dialdehyde presence was included to probe the relevance of these groups in their biological activity on cancer cells.

**Figure 3:**
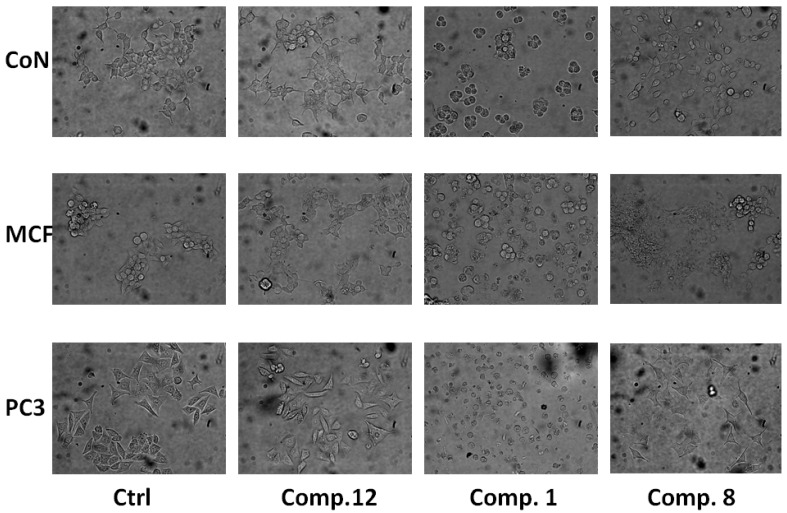
Effect of tested compounds on the morphologies of CoN, MCF-7 and PC-3 cells. Images obtained with an inverted phase contrast microscope (200×) after 24 h exposure of the cells to 50 μM of compounds **1**, **8**, and **12**. (1% ethanol) and similar to control positive (5-fluorouracil, 5-FU).

First, the appearance of morphological changes in the cells treated with 50 μM compound for 24 h was analyzed. Direct observation, using a phase contrast microscope, revealed that the morphologies of PC-3, MCF-7 and CoN cells were severely distorted and cells became rounded after treatment with compounds **1**, **8**, and **12**. Moreover, the cells showed a reduction in number, indicating an increasing progression toward cell death. The control-treated cells (1% ethanol) displayed normal and healthy shapes (Figure 3).

**Figure 4:**
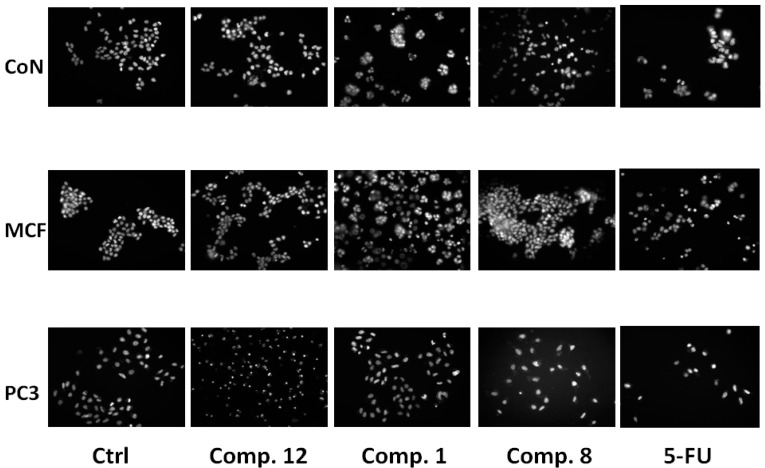
Effect of compounds **1**, **8** and **12** on chromatin condensation and fragmentation. CoN, MCF-7 and PC-3 cells treated with one of the compounds were stained with Hoechst 33342 (200×). Representative photographs presented here show nuclear morphologic changes observed by fluorescent microscopy of the treated cells. As positive control, 5-fluorouracyl (25 μM) was used.

To elucidate whether the compounds reduced cell viability in the cell lines tested by inducing apoptosis (MCF-7, PC-3 and CoN cells), cells treated with each active compound **1**, **8** and **12** were examined after Hoechst 33342 staining. Nuclear changes in PC-3, MCF-7 and CoN cells were observed under a fluorescence microscope (200×, Figure 4). After exposure to the compounds for 24 h, cells treated with compounds **1** and **8** significantly showed chromatin condensation and karyopyknosis compared to control cells (1% ethanol) and similar to control positive (5-fluorouracil, 5-FU) which are typical characteristics of apoptotic process [18]. 

Mitochondria play a crucial role in the apoptotic cascade by serving as a convergent center of apoptotic signals originating from both the extrinsic and intrinsic pathways [19]. Changes induced in the mitochondrial membrane potential (MMP) have been reported previously to represent a determinant in the execution of cell death [20]. As polygodial has been described as an inhibitor of mitochondrial ATPase and also as nonionic surfactant [21,22,23]. This effect may be determinant in cell death, we has analyzed the effect of compounds **1**, **8** and **12** on the mitochondrial membrane potential using flow cytometry with rhodamine 123 stain [24]. As shown in Table 2, the percentage of rhodamine 123 stained-cells were 111.6% ± 8.9%, 77.0% ± 6.5% and 49.1% ± 6.6% in the CoN, MCF-7 and PC-3, respectively, after treatment with compound **1** (50 μM); and 96.0% ± 7.6%, 93.3% ± 7.1% and 116.2 ± 18.8 respectively, after treatment with compound **8**, as compared to 90%–95% in the control cells (1% ethanol). Similar differences were observed after treatments with compounds **1** and **8** at 100 μM.

Table 2 shows that compound **1** increased mitochondrial membrane permeability in cancer cells with a greater effect than compounds **8** and **12**. Moreover the compounds have a weaker effect on mitochondrial membrane potential for the epithelial colon cells, CoN, than in cancer cell lines. Thus, compounds **1** and **8** induced loss of mitochondrial membrane potential, correlated with increased cell death (see Table 2). However, compound **8** has a weaker effect than compound **1**, indicating that the aldehyde group may be important in the loss of mitochondrial membrane potential. Moreover, compound **12** did not change the mitochondrial membrane potential when compared to control cells.

**Table 2:** Compounds **1**, **8** and **12** treatment-induced changes in the mitochondrial membrane permeabilities for CoN, MCF-7 and PC-3 cells. The cells were stained with rhodamine 123, and then analyzed by flow cytometry. The table shows the percentage values of rhodamine 123 stained cells treated without or with compounds **1**, **8** and **12** (50–100 μM) for the different cell lines (* *p <* 0.05 or ** *p* < 0.001 *vs.* control treated cells, assigned 100%). As positive control, FCCP (10 μM) was used.

	CoN		MCF-7		PC-3	
	50 µM	100 µM	50 µM	100 µM	50 µM	100 µM
**Comp. 12**	102.6 ± 9.1	124.9 ± 9.7	111.4 ± 8.9	111.3 ± 9.8	139.1 ± 12.4	149.3 ± 21.3
**Comp. 8**	96.0 ± 7.6	52.3 ± 8.4 *	93.3 ± 7.1	72.3 ± 6.4 *	136.2 ± 18.5	92.8 ± 10.4
**Comp. 1**	49.1 ± 6.6 *	11.8 ± 2.9 **	77.0 ± 6.5 *	19.4 ± 1.5 **	111.6 ± 8.9	11.9 ± 2.4 **
**Control**	93.2 ± 8.7		95.8 ± 9.9		98.1 ± 10.5	
**FCCP**		6.8 ± 1.3 **		7.4 ± 0.4 **		5.2 ± 0.4 **

**Figure 5:**
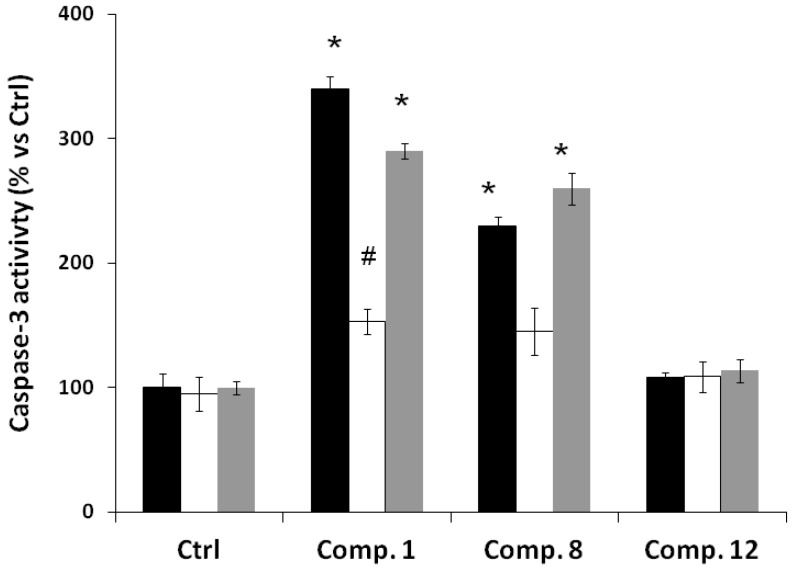
Effect of compounds **1**, **8**, and **12** on caspase-3 activity of MCF-7 (black), CoN (white) and PC-3 (grey) cells. Cells were exposed to compounds at 50 μM for different times. Values are mean ± S.D. (*n* = 3). All data are reported as the percentage change in comparison with the vehicle-treated cells (1% ethanol), which were arbitrarily assigned 100%. # *p* < 0.05, significantly different from the vehicle-treated cells (1% ethanol in medium, that is, compound concentration = 0) and * *p* < 0.001, significantly different from the vehicle-treated cells.

Depletion of mitochondrial membrane potential leads to the release of apoptogenic factors, such as cytochrome c in the apoptotic cascade, and activation of caspases [25,26]. Then we investigated the effects of our compounds on caspase activity. We focused on caspase-3, which is a main executor of apoptosis playing a central role in its biological processing. We analyzed the effect of treatment with the compound **1**, **8** and **12** on caspase-3 activation in normal and cancer cells. As shown in Figure 5, the activation of caspase-3 in cells exposed to compounds **1** and **8** is increased *versus* control-treated cells (1% ethanol). Compound **1** increased the activity of caspase-3 by 3.4 ± 0.1, 1.4 ± 0.1 and 2.8 ± 0.1 times *versus* control cells in MCF-7 (black), CoN (white) and PC-3 (gray) cells, respectively. Compound **8** increased the activity of caspase 3 by 2.4 ± 0.1, 1.3 ± 0.2 and 2.7 ± 0.1 times *versus* control cells in MCF-7 (black), CoN (white) and PC-3 (gray) cells, respectively. Finally, compound **12** did not change the activity of caspase-3 when compared with control cells. 

## 3. Experimental Section

### 3.1. Spectroscopic Analysis

IR spectra were recorded as thin films on a Nicolet 6700 FT-IR spectrometer (Thermo Scientific, San Jose, CA, USA). Frequencies are reported in cm^−1^. ESI-MS/MS data was collected using a high-resolution hybrid quadrupole (Q) and orthogonal time-of-flight (TOF) mass spectrometer (Micromass Q-Tof, Manchester, UK) with constant nebulizer temperature of 80 °C. The ESI source and the mass spectrometer were operated in a negative ion mode, and the cone and extractor potentials were of 10 eV, with a scan range of *m/z* 100–500. The band infused into the ESI source at flow rates of 5 μL·min^−1^ was dissolved in acetonitrile ion-induced dissociation (CID) with argon in the collision chamber. The values expressed are average mass and correspond to the [M-H] (University of Talca). ^1^H, ^13^C, ^13^C DEPT-135, sel. *gs* 1D ^1^H NOESY, *gs* 2D HSQC, and *gs* 2D HMBC spectra were recorded in CDCl_3_ solutions and are referenced to the residual peaks of CHCl_3_ at δ = 7.26 ppm and δ = 77.0 ppm for ^1^H and ^13^C, respectively, on a Bruker Avance 400 Digital NMR spectrometer (Bruker, Rheinstetten, Germany), operating at 400.1 MHz for ^1^H and 100.6 MHz for ^13^C. Silica gel (200–300 mesh, Merck, Santiago, Chile) was used for column chromatography (C.C.) and HF-254 silica gel plates were used for TLC. TLC spots were detected by heating after spraying with 25% H_2_SO_4_ in H_2_O.

### 3.2. Plant Material

The stem bark of *D. winteri* adult trees was collected from the Malleco Province (Chile), in February 2010. A voucher specimen (N° Dw-10113) was deposited at the Herbarium of the Natural Products Laboratory, “Dr. Herbert Appel A.”, Department of Chemistry, Universidad Técnica Federico Santa María, Valparaíso, Chile. Fresh bark was carefully washed with abundant distilled water to remove any residue. Afterwards, it was dried in an oven at 35 °C to a constant weight. Once dried, it was stored in hermetically sealed plastic containers at 4 °C.

### 3.3. Isolation of Natural Compounds **1**–**4**

The natural drimanes polygodial (**1**), isopolygodial (**2**), drimenol (**3**), confertifolin (**4**), and isodrimenin (**5**) (Figure 1) were isolated from the dichloromethane extract of *D. winteri* (Winteraceae) bark. The extraction methodology and isolation of pure compounds was performed according to reported procedures [27,28]. Compounds **1**–**5** were identified by melting point, optical rotation, and spectroscopic data, including ^1^H- and ^13^C-NMR and comparisons with data reported in the literature [16,28,29]. 

### 3.4. Preparation of Polygodial Derivatives **6**–**8** and Drimenol Derivatives **9**–**12**

Compounds **6**–**8** were synthesized by treating polygodial (**1**) using different protocols reported in the literature [16,30,31]. Compounds **9**–**12** were synthesized by treating drimenol (**3**) using different protocols reported in the literature [15,28,29]. The synthesis of compounds **11** and **12** is described below:

*Synthesis of (5S,7S,8R,9S,10S)-4,4,8β,10β-tetramethyldecahydronaphtho[8,9-α]oxiren-7-yl acetate* (**11**) *from* **10**. Compound **10** (1.0 g, 3.99 mmol) was treated in CH_2_Cl_2_ (30 mL) with *m*-CPBA (0.94 g, 5.4 mmol). Then the mixture was stirred for 1 h at room temperature. After this, the solution was taken up in CH_2_Cl_2_ (20 mL) and then washed with saturated NaHCO_3_ solution. Later, the organic layer was dried over MgSO_4_, filtered, and evaporated. Then it was absorbed on silica, and subjected to C.C. eluting with mixtures of petroleum ether/EtOAc of increasing polarity (20.0:0.0→18.0:2.0) to give a white solid identified as epoxide **11** (0.74 g, 68.0%.); m.p: 68.5–70.0 °C. ${\left[\alpha \right]}_{D}^{17}$ = +0.36° (c = 14.58, CHCl_3_). IR (cm^−1^): 1779; 1452; 1254. ^1^H-NMR: 5.05 (t, 1H, *J* = 8.8 Hz, H-7); 2.52 (s, 1H, H-9); 2.09 (s, 3H, CH_3_); 1.88 (m, 1H, H-6α); 1.60 (m, 2H, H-5 and H-6 β); 1.47 (m, 4H, H-1 and H-2); 1.24 (s, 3H, H-11); 1.18 (m, 2H, H-3); 1.06 (s, 3H, H-14); 0.84 (s, 3H, H-12); 0.79 (s, 3H, H-13). ^13^C-NMR: 170.4.2 (CH_3_CO); 71.5 (C-7); 70.7 (C-9); 58.9 (C-8); 4154 (C-5); 39.9 (C-3); 36.3 (C-1); 34.5 (C-5); 33.0 (C-10); 32.5 (C-13); 25.0 (C-12); (CH_3_CO); 21.1 (C-2); 19.5 (C-14); 18.2 (C-11). HREIMS: M+H ion *m*/*z* 267.1882 (calcd. for C_16_H_26_O_3_: 266.3819).

*Synthesis of 8α,9α-epoxi-11-nordriman-7-one* (**12**) *from* **11**. Compound **11** (0.25 g, 1.12 mmol) was treated with a solution of Na_2_CO_3_ in MeOH (50 mL, 15%). Then the mixture was oxidized with PDC (1 g, 2.66 mmol) and was stirred for 1.5 h at room temperature. After this, the solution was taken up in CH_2_Cl_2_ (20 mL) and washed with saturated NaHCO_3_ solution. The organic layer was dried over MgSO_4_, filtered, and evaporated. Then, it was absorbed on silica, and subjected to column chromatography eluting with mixtures of petroleum ether/EtOAc of increasing polarity (20.0:0.0→18.0:2.0) to give a white solid identified as epoxide **12** (0.10 g; 0.26 mmol, 40.0%), m.p: 38–39 °C. ${\left[\alpha \right]}_{D}^{17}$ = −0.16° (c = 2.0, CHCl_3_). IR (cm^−1^): 1706, 1464, 1117. ^1^H-NMR: 2.86 (s, 1H, H-9); 2.37 (dd, 2H, H-6); 2.05 (m, 2H, H-1); 1.89 (dd, 3H, H-2 and H-5 ), 1.34 (s, 3H, H-11); 0.92 (s, 3H, H-14); 0.85 (s, 3H, H-12); 0.81 (s, 3H, H-13). ^13^C-NMR: 207.5 (C-7); 71.4 (C-9); 60.3 (C-8); 41.1 (C-10); 41.0 (C-3); 36.4 (C-5); 34.9 (C-4); 34.4 (C-2); 32.7 (C-13); 32.1 (C-12); 20.0 (C-1); 18.3 (C-6); 17.0 (C-14); 15.5 (C-12). HREIMS: M+H ion *m/z* 223, 1618 (calcd. for C_14_H_22_O_2_: 222.3286).

### 3.5. Cell Lines

The experimental cell cultures were obtained from the American Type Culture Collection (Rockville, MD, USA). MCF-7 cells (breast cancer cell line), PC-3 and DU-145 (prostate cancer cell lines), and human colon epithelial cells CCD 841 CoN (non-tumoral cell line) were grown in Dulbecco’s modified Eagle’s medium (DMEM) containing 10% FCS, 100 U/mL penicillin, 100 μg/mL streptomycin, and 1 mM glutamine. Cells were seeded into 96 well microtiter plates in 100 μL at a plating density of 5 × 10^3^ cells/well. After 24 h incubation at 37 °C under a humidified 5% CO_2_ atmosphere to allow cell attachment, the cells were treated with different concentrations of drugs and incubated for 72 h under the same conditions. Stock solutions of compounds were prepared in ethanol and the final concentration of this solvent was kept constant at 1%. Control cultures received 1% ethanol alone. 

### 3.6. Cell Viability

The sulforhodamine B assay was used according to the method of Skehan *et al.* [32]. Briefly, the cells were set up at 3 × 10^3^ cells per well of a 96 200 μL well, flat-bottomed, microplate. Cells were incubated at 37 °C in a humidified 5% CO_2_/95% air mixture and treated with the compounds at different concentrations for 72 h. At the end of drug exposure, cells were fixed with 50% trichloroacetic acid at 4 °C. After washing with water, cells were stained with 0.1% sulforhodamine B (Sigma-Aldrich, St. Louis, MO, USA), dissolved in 1% acetic acid (50 μL/well) for 30 min, and subsequently washed with 1% acetic acid to remove unbound stain. Protein-bound stain was solubilized with 100 μL of 10 mM unbuffered Tris base, and the cell density was determined using a fluorescence plate reader (wavelength 540 nm). Values shown are the mean ± SD of three independent experiments in triplicate. The software used to calculate the IC50 values was GraphPad (GraphPad Software, San Diego, CA, USA).

### 3.7. Morphological Assessment of Cell Apoptosis

Morphological changes in the nuclear chromatin of cells undergoing apoptosis were revealed by a nuclear fluorescent dye, Hoechst 33342. Briefly, on 24-well chamber slides, 1 × 10^4^ cells/mL were cultured and exposed to compounds for 24 h. The control group was also exposed to 1% ethanol. The cells were washed twice with phosphate buffer solution, fixed with 3.7% formaldehyde, and washed again with phosphate buffer solution. Following the addition of 1 μM Hoechst 33342 (Sigma-Aldrich, Santiago, Chile), they were reacted in a dark room at room temperature for 30 min. After washing, they were examined under an immunofluorescence microscope (IX 81 model inverted microscope, Olympus, Santiago, Chile).

### 3.8. Analysis of Mitochondrial Membrane Permeability

Rhodamine 123, a cationic, voltage-sensitive probe that reversibly accumulates in mitochondria, was used to detect changes in transmembrane mitochondrial membrane potentials. Exponentially growing cells were incubated with the compound as indicated in the figure legends. Cells were labelled with 1 μM rhodamine 123 at 37 °C in cell medium for 60 min before terminating the experiment. Cells were detached from the plate, after washing with ice cold PBS, and the samples were analyzed by flow cytometry. Data are expressed in percentage of cells with rhodamine 123 [24].

### 3.9. Caspase 3 Activity Assay

Caspase activity was measured using a colorimetric assay [33]. Briefly, the cells exposed to compounds were collected by centrifugation at 1000 rpm and lysed with lysis buffer (1% Triton X-100, 0.32 M sucrose, 5 mM EDTA, 10 mM Tris–HCl, pH 8.0, 2 mM dithiothreitol, 1 mM PMSF, 1 g/mL aprotinin, 1 mg/mL leupeptin). Thereafter, the lysates were transferred to wells in a 96-well microplate and were incubated with DEVD-pNA (final concentration 200 μM) specific for caspase-3/7, at 37 °C for 1 h. The intensity of the developed color was read at 405 nm in a microplate reader (SpectraMax, Winooski, VT, USA). The results are expressed as percentages of the control level.

### 3.10. Statistical Analysis

Data shown in tables are average results obtained by means of three or 10 replicates and are presented as average ± standard errors of the mean (SEM). Data were subjected to analysis of variance (ANOVA) with significant differences between means identified by GLM procedures. Results are given in the text as probability values, with *p* < 0.05 adopted as the criterion of significance. Differences between treatment means were established with a Student–Newman–Keuls (SNK) test. The EC_50_ values for each activity were calculated by PROBIT analysis based on percentage of mortality obtained at each concentration of the samples. EC_50_ is half maximal effective concentration. Complete statistical analysis was performed by means of the MicroCal Origin 6.0 statistics and graphs PC program.

## 4. Conclusions

The results show that only polygodial (**1**), isodrimenin (**5**), aldehyde **8** and compounds **9** and **12** had IC_50_ values lower than 200 µM against a panel of cancer cell lines. We found that a higher concentration of polygodial (**1**) decreases the cell viability of DU-145 and PC-3, MCF-7. Drimenol (**3**) and confertifolin (**4**) have an effect on the cell lines at higher concentrations, but only in PC-3 did they show activity against tested cells, DU-145, at lower concentrations. Compounds **1** and **8** showed a good apoptotic response against prostate cancer cell cultures. Moreover, compounds **1** and **8** also depleted the mitochondrial membrane potential and increased caspase-3 activity. Compound **12** neither changed the mitochondrial membrane permeability nor the activity of caspase-3, suggesting that the presence of a dialdehyde had relevance for the apoptotic activity in the cell lines studied.
